# FEAR OF FAILING: PERSPECTIVES ON HOME- AND COMMUNITY-BASED SUPPORT SERVICES FOR RURAL CAREGIVERS

**DOI:** 10.1093/geroni/igad104.2975

**Published:** 2023-12-21

**Authors:** Elizabeth Marfeo, Elizabeth Chamberlin, Maria Venegas, Lauren Moo

**Affiliations:** Tufts University, Medford, Massachusetts, United States; US Department of Veterans Affairs, Bedford, Massachusetts, United States; US Department of Veterans Affairs, Bedford, Massachusetts, United States; Veterans Health Administration, Bedford, Massachusetts, United States

## Abstract

Many older adults rely on family caregivers not only for day-to-day assistance, but also to help navigate the healthcare system and attend medical visits. Finding, scheduling, arranging transportation for, and attending medical visits involves considerable planning, adaptability to a broad array of contextual factors, and can often cause disruption in day-to-day routine of both the family caregiver and the older adult. For Veterans living in rural areas, limited VA and community resources may magnify these challenges leading to higher caregiving burden and negative health and quality of life outcomes for both the Veteran and caregiver. The aim of this study was to conduct in-depth interviews to better understand key contextual factors related to the logistics of managing and attending medical visits among family caregivers of Veterans living in rural communities (N = 19, mean age 65). A clear disconnect emerged between caregiver needs and (1) access to community support services and (2) effective utilization of existing services. Seventy-nine percent of the caregivers reported living with the Veteran and receiving no home services. Interviews revealed both the caregiver and Veteran having significant, complex healthcare needs. Caregivers described fears of their own health negatively affecting their ability to continue providing sufficient supports for their loved one, lack of resources for home management needs, and limited access to transportation assistance. Findings from this study are important for informing continued development and evaluation of caregiver support programs nationwide, especially among those living in rural communities.

